# Numerical simulation of strain-adaptive bone remodelling in the ankle joint

**DOI:** 10.1186/1475-925X-10-58

**Published:** 2011-07-05

**Authors:** Anas Bouguecha, Nelly Weigel, Bernd-Arno Behrens, Christina Stukenborg-Colsman, Hazibullah Waizy

**Affiliations:** 1 Institute of Metal Forming and Metal-Forming Machines, Leibniz Universität Hannover, An der Universität 2, 30823 Garbsen, Germany; 2 Department of Orthopaedic Surgery, Hannover Medical School, Anna-von-Borries-Straße 1-7, 30625 Hanover, Germany

## Abstract

**Background:**

The use of artificial endoprostheses has become a routine procedure for knee and hip joints while ankle arthritis has traditionally been treated by means of arthrodesis. Due to its advantages, the implantation of endoprostheses is constantly increasing. While finite element analyses (FEA) of strain-adaptive bone remodelling have been carried out for the hip joint in previous studies, to our knowledge there are no investigations that have considered remodelling processes of the ankle joint. In order to evaluate and optimise new generation implants of the ankle joint, as well as to gain additional knowledge regarding the biomechanics, strain-adaptive bone remodelling has been calculated separately for the tibia and the talus after providing them with an implant.

**Methods:**

FE models of the bone-implant assembly for both the tibia and the talus have been developed. Bone characteristics such as the density distribution have been applied corresponding to CT scans. A force of 5,200 N, which corresponds to the compression force during normal walking of a person with a weight of 100 kg according to Stauffer et al., has been used in the simulation. The bone adaptation law, previously developed by our research team, has been used for the calculation of the remodelling processes.

**Results:**

A total bone mass loss of 2% in the tibia and 13% in the talus was calculated. The greater decline of density in the talus is due to its smaller size compared to the relatively large implant dimensions causing remodelling processes in the whole bone tissue. In the tibia, bone remodelling processes are only calculated in areas adjacent to the implant. Thus, a smaller bone mass loss than in the talus can be expected. There is a high agreement between the simulation results in the distal tibia and the literature regarding.

**Conclusions:**

In this study, strain-adaptive bone remodelling processes are simulated using the FE method. The results contribute to a better understanding of the biomechanical behaviour of the ankle joint and hence are useful for the optimisation of the implant geometry in the future.

## Background

Arthrodesis is a preferred and most used operative therapy in advanced symptomatic arthrosis of the ankle [1]. The objective is pain relief with a stable osseous fusion. Disadvantages are the loss of movement in the joint accompanied by an increased mobility in the transversal joint (Chopart joint) as a compensation reaction. This can lead to secondary overloading and arthrosis. The activity of the patient is significantly limited by arthrodesis. Further disadvantages are the risk of non fusion as well as the long rehabilitation time [2-6].

**Figure 1:**
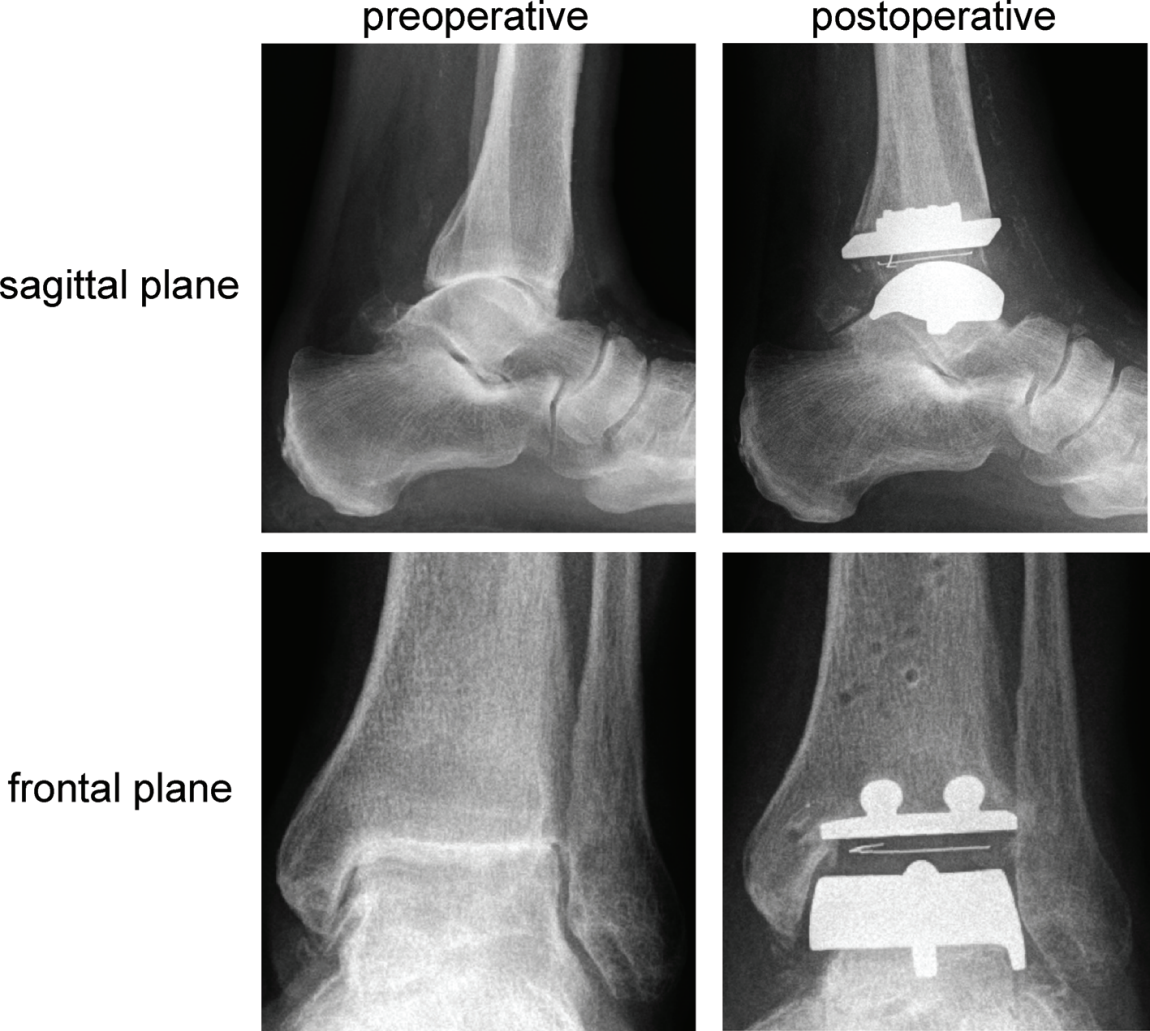
**Radiographs of a left arthrotic ankle joint pre-and postoperative in sagittal and frontal plane**.

Total ankle replacement (TAR) is an often used alternative procedure in advanced arthrosis of the ankle. Preoperative and postoperative radiographs of a left arthrotic ankle treated with the three-component system S.T.A.R.^® ^(Small Bone Innovations, Donaueschingen/Germany) are shown in Figure 1.

The advantage of TAR is the maintenance of the ankle motion. Therefore, TAR should reduce the load on the adjacent joints and prevent the development of secondary arthrosis [7]. However, the development of TAR is lagged behind that of the hip and knee [8]. Difficulties are the smaller joint size [9] and the comparatively higher stresses applied to the ankle joint resulting from higher compression forces [10-14] and torques [15,16]. Furthermore, patients requiring TAR are generally younger and therefore more active [1,2].

**Figure 2:**
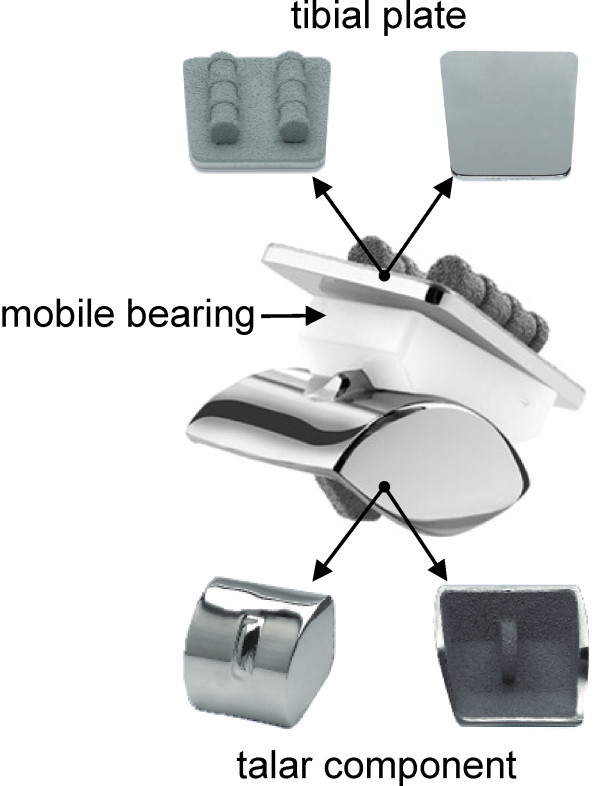
**S.T.A.R.^® ^system **[19].

The clinical results of TAR improved in recent years [17]. Nowadays, there are 82% of good to very good results with ankle joint prostheses according to AOFAS scores. With arthrodesis there are 72% of good to very good results achieved with the Mazur ankle score [18]. One reason for the improvement was the development of the modern three-component prostheses [17]. The actual TAR systems consist of three components [19]: a metallic baseplate fixed to the tibia, a domed or condylar shaped metallic component that resurfaces the talus, and a mobile bearing inlay, which consists of ultra-high molecular weight polyethylene (Figure 2).

Because of their full congruency without restriction of rotational motion, the modern three-component designs have mechanical and kinematical advantages compared to two-component designs. The rotational stresses at the bone-prosthesis interface are reduced by this development. Independent from the exact design, the 5-year survival rate of the third generation endoprostheses is up to 90% [20].

However, the results still indicate inferior patient satisfaction, compared to knee and hip arthroplasty, as mentioned previously. Aseptic loosening is the main reason for early failure of TAR [21]. It results from stress shielding caused by the implant. The difference in mechanical properties of the bone tissue and the prosthesis and the changed bone loading condition due to TAR promote stress shielding. Bone tissue is in a permanent state of resorption and formation. Bone acts like a technical controller on load changes [22]. Set point is the elastic deformation, which is regulated by a variation of the amount of bone tissue. Accordingly, a load increase leads to an increase in the elastic deformation and to the formation of new bone tissue. Contrarily, a load reduction leads to bone resorption [23]. The implantation of an artificial joint leads to a change in biomechanics. This can lead to intense bone resorption around the implant and can cause the migration or loosening of the implant.

In order to increase long-term stability and to avoid implant loosening, it is important to estimate the adaptive bone remodelling prior to implantation in preclinical studies.

In our previous studies, bone remodelling processes were successfully simulated for canine [24] and human [25,26] femora after total hip replacement (THR). The influence of the boundary conditions were also investigated [27]. Furthermore, the significance of implant materials [28,29] and implantation techniques [30,31] of the remodelling processes in the femur were analysed. Recently, bone remodelling in the acetabulum was simulated as well [32].

In general, bone remodelling was calculated mainly in the femur [24-31,33-38]. To our knowledge, there exist no simulations of the bone remodelling in the ankle joint.

FE simulations concerning TARs are quite rare. The following section provides an overview of simulations at the ankle joint found in the literature.

In the middle of the 1980s Falsig et al. [39] employed a 3D finite element stress analysis to calculate the stresses in the distal tibia. The goal was to reduce the cement-bone-interface and the stresses in the bone by varying the shape and the material of the tibial component. Prostheses with metal backing are advantageous compared to PE components without metal backing. Furthermore, long stems for the implant fixation proved unfavourable as well.

The stresses and wear of the PE inlay of TARs was examined by FEA from the research group of McIff [40,41]. In the first study a two component design (AGILITY™) and a three component design (S.T.A.R.^®^) were compared. For the AGILITY™ system the loss of congruency between the PE inlay and the talar component resulted in high contact and internal stresses due to point and line contact. In a second study the design of the inlay and of the talar component was varied. Apparent insignificant geometrical specifications had an important influence on the contact mechanism.

A recent study of Espinosa et al. [42] confirmed the result of the first examination of McIff et al. [40]. The research group around Galik [43] examined the PE wear and varied the thickness of the PE inlay.

More complex models were established by Reggiani et al [44] and Anderson et al. [45]. Reggiani calculated the contact pressure on the components. They computed average values of 6.4 MPa for the tibial component and 10.3 MPa for the talar component [44].

Anderson et al. [45] developed a complex FE model for the simulation of the contact pressure distribution in the healthy ankle joint. Recently, Anderson et al. [46] performed a FEA with a new implant for defect resurfacing of the talus according clinical feasibility.

The aim of this study is to calculate the strain-adaptive bone remodelling after TAR by means of the finite element method (FEM).

## Methods

### Geometry modelling

First, FE models of the tibia and the talus should be built. Therefore, models of the intact bone and the bone provided with the implant are required. In order to obtain geometrical data, a left ankle joint from Sawbones (Malmö/Sweden) was optically measured with the 3D coordinate measurement system ATOS II (GOM mbH, Braunschweig/Germany). The bones from Sawbones were used to obtain a standard geometry model of the ankle joint and to eliminate patient-individual anatomical characteristics.

The digitalised STL data of the tibia and the talus were reconstructed by means of the 3D software Mimics (Materialise, Leuven/Belgium). CT scans (Philips Brilliance CT 64) of 20 cadaver ankle joints were performed to define the density distribution throughout the bone on the basis of the gray values. Accordingly, the tibial bone model was divided into three different density areas: cortical bone in the outer layer (1.7 g/cm^3^), cancellous bone in the inner layer (0.5 g/cm^3^) and an interface layer between cortical and cancellous bone (1.0 g/cm^3^). The talus was divided into only two different density areas namely cortical bone (1.7 g/cm^3^) and cancellous bone (0.5 g/cm^3^). An interface layer has not been observed on the CT scans.

The bone tissue was modelled with homogeneous elastic properties. On the basis of the density values the Young's modulus was determined according to Equation 1 [47].(1)

In order to build the FE models of the bone-implant assembly, the prosthesis was integrated into the previously described bone models via the pre-processor software HyperMesh (Altair Engineering GmbH, Böblingen/Germany). The cementless three component S.T.A.R.^® ^system was used, which is the most-implanted ankle joint system in Europe [7] (Figure 2).

The alignment of the implant was defined based on surgical experience.

**Figure 3:**
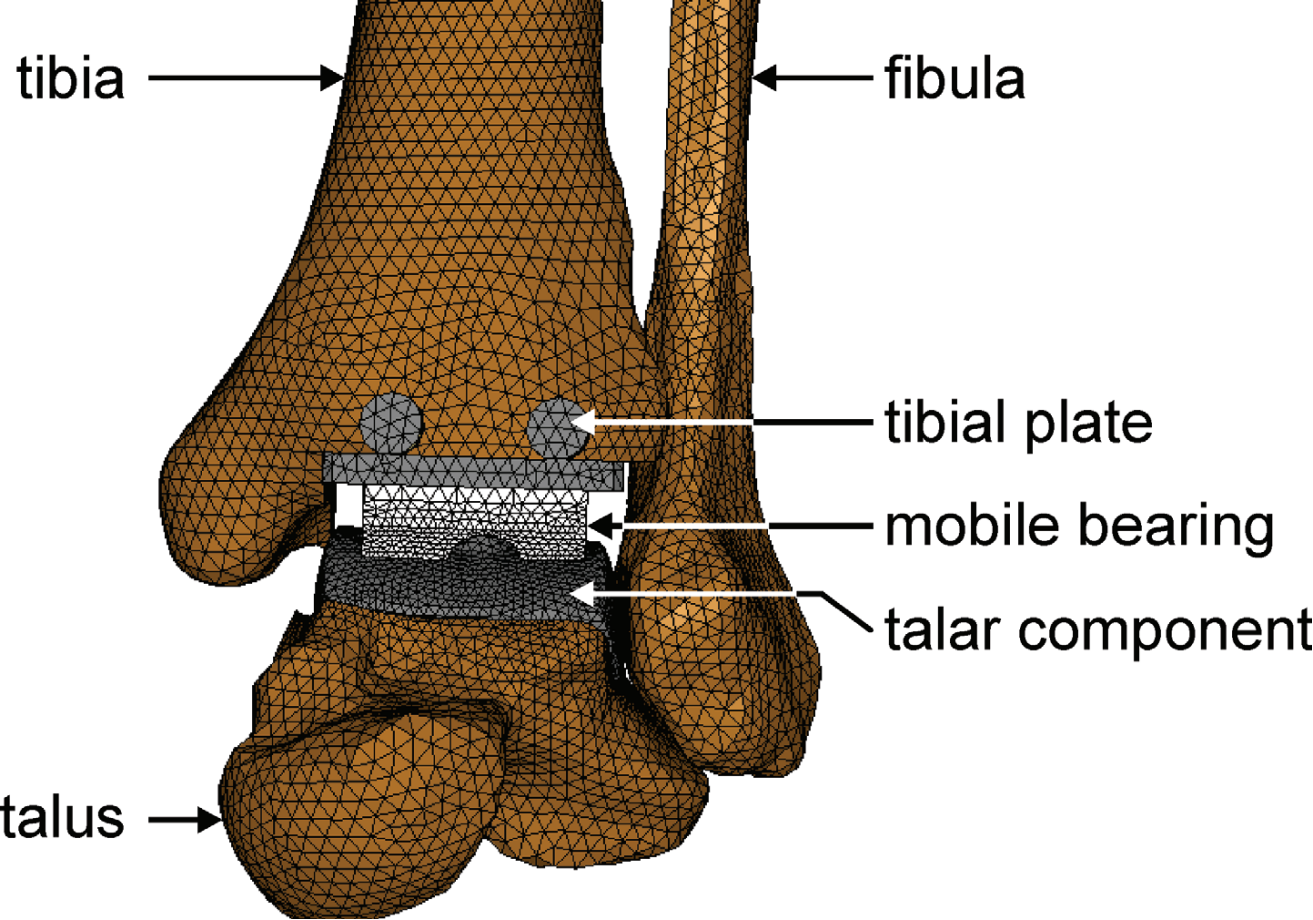
**FE solid model of the prosthetic ankle joint**.

After the virtual implantation of the tibial and the talar component in the bone models, they were meshed using four-noded tetrahedral elements via the pre-processor HyperMesh. The FE solid model of the whole ankle joint with the endoprosthesis is shown in Figure 3.

For an optimal force transmission from the implant to the bone and for the modelling of the cementless fixation of the implant, a consistent meshing method has been used to realise these requirements. In addition, the models of the tibia and talus in the physiological state without implant already provide the implant geometry. Errors resulting from density transmission inaccuracies are excluded a priori.

The prosthesis is made of a cobalt-chromium-molybdenium alloy with titanium plasma spray coating. In the FE modelling a homogeneous, isotropic material law was used for the prosthesis (*E *= 210,000 N/mm^2^, *ν *= 0.3). The coating was considered using proper friction coefficients between the bone and the implant surface.

### Loads and boundary conditions

**Figure 4:**
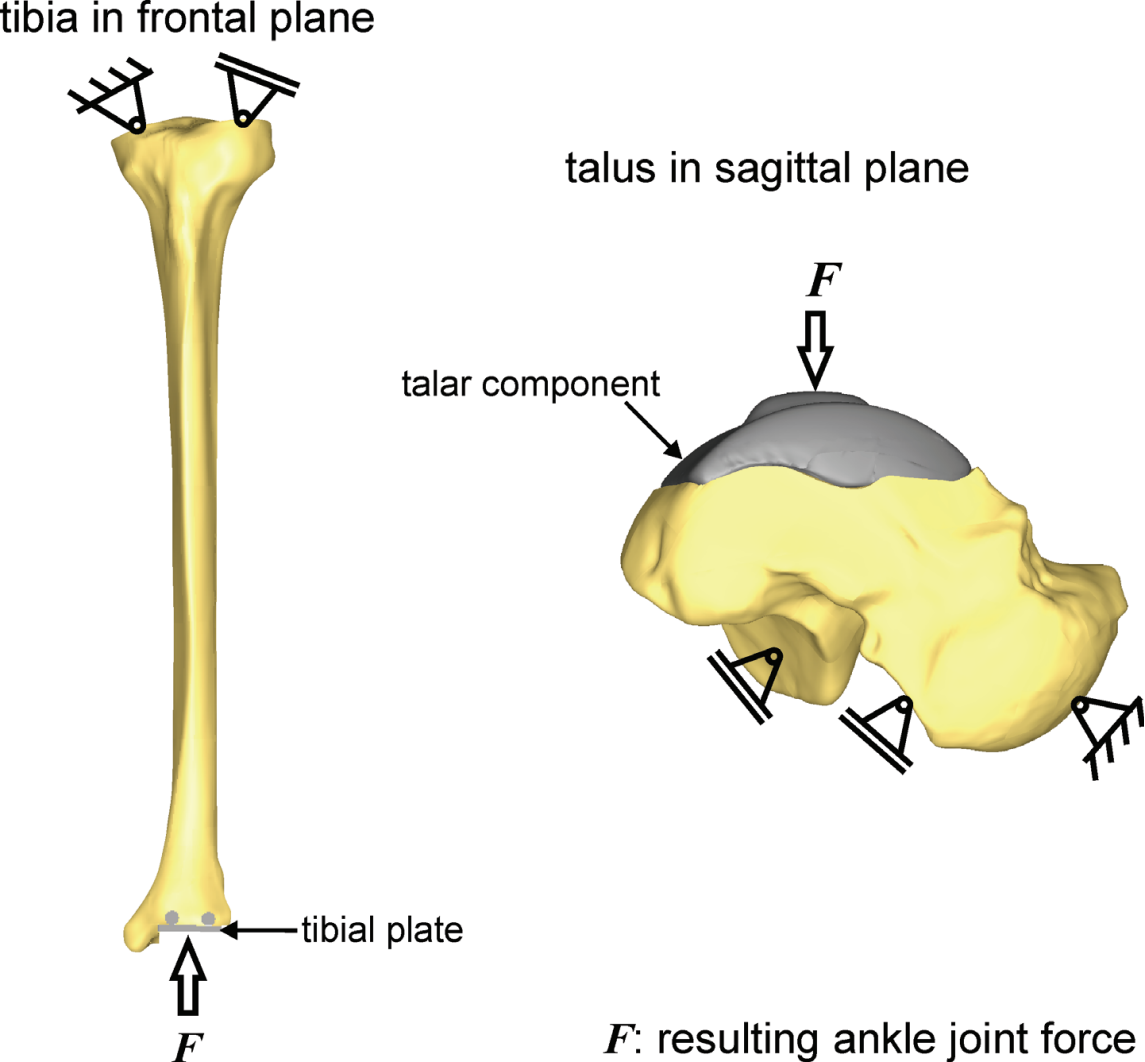
**Boundary conditions for the tibia and the talus**.

Subsequent to modelling, both loads and boundary conditions were defined. The tibia was constrained at the proximal plateau as illustrated in Figure 4. Fixed bearings were used. The load was applied distally. Contrarily, the talus was constrained distally on the subtalar joints by the use of fixed bearings and the force was applied proximally (Figure 4).

The load was taken from the study of Stauffer et al. [10], in which a load of 5.2 times the body weight had been calculated for the normal ankle joint during the stance phase of gait. A person of 100 kg weight was assumed. Consequently, a static force *F *of 5,200 N was applied to the tibia and the talus by spreading it equally on ten nodes.

### FE simulation of the bone remodelling

**Figure 5:**
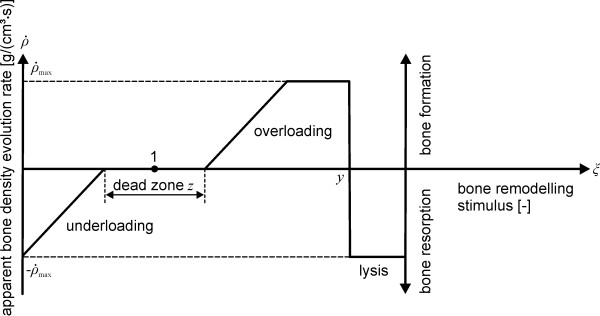
**Bone adaptation law of Bouguecha et al. **[32]**used in the simulation**. In this figure, the apparent bone density evolution rate *ṗ* in dependency of the bone remodelling stimulus *ξ *is illustrated. Bone resorption occurs in an underloading condition when the bone formation rate is negative. Formation of new bone tissue occurs in an overloading condition and a positive bone formation rate. Within a dead zone *z*, a change of the bone remodelling stimulus does neither lead to formation nor to resorption of bone tissue. After exceeding a certain stimulus *y*, bone tissue is overloaded to such an extent, that lysis of bone tissue occurs.

As a next step the simulation was carried out using the FE solver MSC.MARC (MSC.SOFTWARE Corp.). This was done for the talus and the tibia, separately. The same force and force application points were used in both simulations. Furthermore, the bone adaptation law of Bouguecha et al. [32] was applied (Figure 5).

The bone formation rate  changes in dependency of the bone remodelling stimulus *ξ*. The stimulus is defined by the ratio of the actual strain energy per unit of mass in the periprosthetic bone *S*_pro _to that in the physiological bone *S*_ref _(Equation 2).(2)

The strain energy density per unit of mass is calculated according to Equation 3.(3)

Herein, *ε* represents the strain vector and *σ*^*T *^the transposed stress vector.

The used bone adaptation law is a modification of the law of Huiskes [34]. A limitation of the bone formation rate has been introduced. It is assumed, that severe overloading causes lyses of bone tissue and no unlimited bone formation.

On the basis of a constant element volume, the density values of each element were recalculated in every increment. The apparent bone density evolution rate  was calculated depending on the stimulus for the bone remodelling. After the convergence of the calculation at a constant density value, a balance is adjusted between resorption and forming of bone tissue. The remodelling of bone tissue can be indicated according to the density distribution in the tibia and the talus at the end of the simulation.

## Results

### Bone remodelling in the tibia

**Figure 6:**
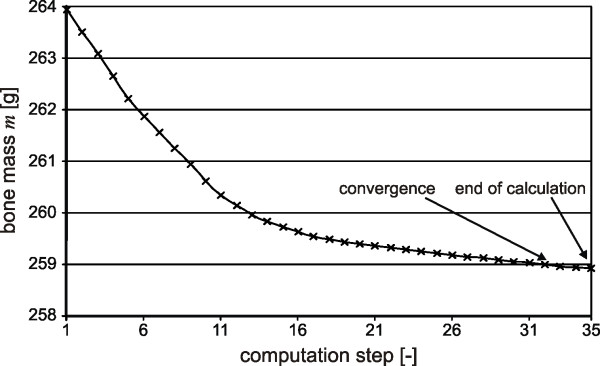
**Bone mass change in the tibia over the computation steps**. In this figure, the bone mass loss in dependency of the computation steps is shown. At the beginning of the calculation there is a great decline in the bone mass. After convergence of the bone mass after a certain computation step, the calculation is finished.

According to the numerical calculations, a total bone mass loss of 2% in the whole prosthetic tibia can be expected. The progress of the mass loss in the tibia is presented in Figure 6. The initial state in the simulation (computation step 1) corresponds to the medical situation directly after TAR, while the stationary final state (computation step 35) corresponds to the clinical long-term situation.

**Figure 7:**
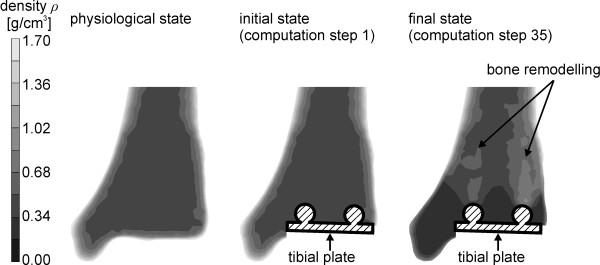
**Density distribution in the distal tibia**. The density distribution in frontal section for the physiological (left image) and periprosthetic states of the tibia (middle and right image) is shown. Density values range from 0.00 up to 1.70 g/cm^3^. Comparing the final and the initial states of the periprosthetic tibia bone remodelling areas can be determined according to the density changes which are exemplarily marked for bone formation in the right image.

Bone remodelling processes in the prosthetic tibia only occur in the distal epiphysis, where the implant is embedded. A change in density distribution is not calculated within the proximal tibia. Subsequently, the post-convergence distribution of the bone density for the distal tibia is shown in Figure 7.

To the left image of Figure 7, the density distribution in the physiological state without implant is shown, whereas on the two right images, the density distribution of the prosthetic ankle is represented.

An increase in density is to be expected above both anchoring bolts, while a decrease in density is calculated centrally above the tibial plate and in the medial malleolus.

### Bone remodelling in the talus

**Figure 8:**
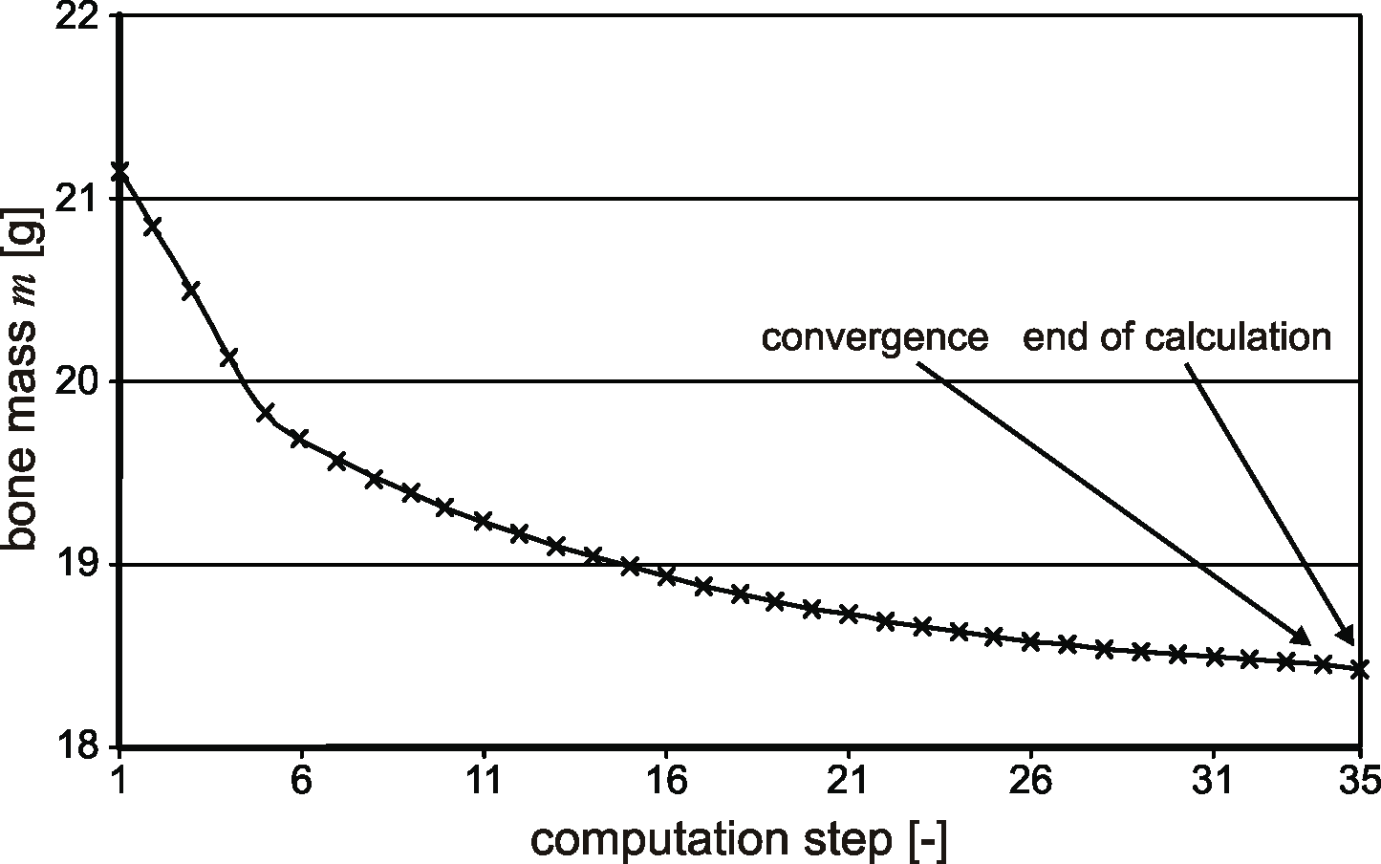
**Bone mass change in the talus over the computation steps**.

A total bone mass loss of 13% has been estimated for the talus. In Figure 8 the progress of the mass loss in the talus is presented.

**Figure 9:**
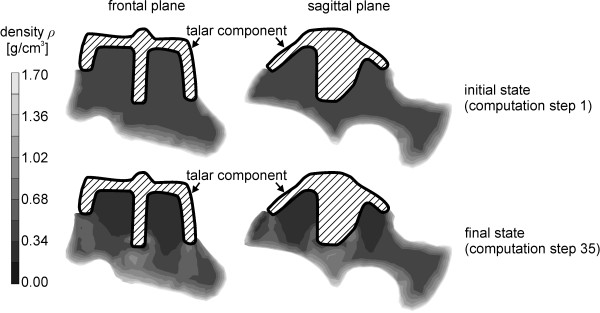
**Density distribution in the talus in frontal and sagittal plane**.

The high bone mass loss is expected due to the small size of the bone and the dimensions of the implant. Bone remodelling processes occur in large regions all over the talus. The density distribution in the whole talus in frontal and sagittal plane is illustrated in Figure 9. The upper images illustrate the initial state and the lower images the final state after convergence of the calculation.

The bone mass loss is observed in the regions beneath the implant. A considerable increase in density is expected according to the FE analysis beneath the central fin of the component.

## Discussion

The number of TARs is constantly increasing [7]. Thomas and Daniels [48] pointed out, that beside good clinical results with modern three-component systems, no statements can be made about the long-term stability of the implants.

This makes it even more important to simulate the bone remodelling in order to estimate the clinical performance of the endoprostheses prior to implantation.

In this work, a 3D FE simulation of the bone remodelling processes in the tibia and the talus was performed. The bone adaptation law used in the study has been calibrated for the hip joint via DEXA investigations in recent studies [26]. The calculated bone mass loss was in good agreement to the conclusions of the DEXA analysis. The application of the adaptation law to the ankle joint is considered to be adequate.

Nevertheless, every FE simulation has to be validated in order to proof the accuracy of the calculation result. Therefore, further DEXA investigations of the ankle joint after TAR are still required.

The simulation was done using homogeneous elastic properties for the bones, although bone tissue consists of cortical and cancellous bone with complex trabecular architecture. This simplification was made due to the observation on the CT scans, where the patterns of the cancellous bone were not clearly visible. A nearly homogeneous density distribution for the cancellous and cortical bone in the talus has been observed. For the tibia, a third density area was added between cortical and cancellous bone to reflect the observations on the CT scans.

Finally, first results of the 3D FE simulation concerning the strain-adaptive bone remodelling processes in the whole ankle joint were presented in this study.

Good agreement was achieved between the simulation result in the tibia and the changes in density distribution after TAR described in the literature. An increase in density has been observed above the anchoring bolts of the implant and a density decrease was observed centrally above the tibial plate [2]. This confirms the previously described areas of strain-adaptive bone remodelling. The fixation of the tibial component is achieved with two anchoring bolts. This results in force transmission from the two bars into the bone. This may lead to stress shielding between the anchoring bolts and above the tibial plate which has also been observed from Hintermann [2].

In the simulated model the fibula was disregarded because there is no direct fixation of an implant at the fibula. Furthermore, it does not contribute to the load distribution from the tibia to the talus. The fibula is irrelevant for bone remodelling processes in the ankle joint and hence neglecting its role is justified. Other researchers who examined the ankle joint by FEM [42-48] disregarded the fibula as well.

Another simplification was the use of only one static load case. In reality more complex loading conditions can be expected. Moreover, the muscle forces acting on the ankle joint were disregarded due to the lack of experimental study. For future investigations, force patterns, derived from multibody simulations, should be incorporated into the FE calculation to consider the whole gait cycle and in order to examine the influence of the muscle forces. Furthermore migration processes of the implants can be calculated by coupling the multibody simulation and the FE calculation.

Further investigations are planned regarding the previously described limitations.

## Conclusions

To our knowledge the first finite element simulation of strain-adaptive bone remodelling of the ankle joint is presented in this study. High agreement between the simulation and the clinical results were shown in a qualitative comparison between the calculated bone remodelling in the distal tibia and the reported radiographic changes in literature [2]. On the basis of the calculated density distributions, the design of total ankle prostheses can be evaluated and optimised.

## Competing interests

The authors declare that they have no competing interests.

## Authors' contributions

AB designed the study, developed the numerical computation method of the bone remodelling and corrected the manuscript. NW constructed the finite element models, performed the calculations and prepared the manuscript. BAB and CSC designed the study concept from a technical and medical perspective. HW designed the study from the medical perspective and also prepared the manuscript. All authors read and approved the final manuscript.
